# Comprehensive Analysis of Therapy-Related Messenger RNAs and Long Noncoding RNAs as Novel Biomarkers for Advanced Colorectal Cancer

**DOI:** 10.3389/fgene.2019.00803

**Published:** 2019-11-20

**Authors:** Jibin Li, Siping Ma, Tao Lin, Yanxi Li, Shihua Yang, Wanchuan Zhang, Rui Zhang, Yongpeng Wang

**Affiliations:** ^1^Department of Colorectal Surgery, Liaoning Cancer Hospital, Cancer Hospital of China Medical University, Shenyang, China; ^2^China Medical University, Shenyang, China

**Keywords:** long noncoding RNA, snoRNAs, prognostic markers, expression profiling, protein–protein interaction analysis, co-expression analysis, colorectal cancer

## Abstract

Colorectal cancer (CRC) is one of the most common types of human cancers. However, the mechanisms underlying CRC progression remained elusive. This study identified differently expressed messenger RNAs (mRNAs), long noncoding RNAs (lncRNAs), and small nucleolar RNAs (snoRNAs) between pre-therapeutic biopsies and post-therapeutic resections of locally advanced CRC by analyzing a public dataset, GSE94104. We identified 427 dysregulated mRNAs, 4 dysregulated lncRNAs, and 19 dysregulated snoRNAs between pre- and post-therapeutic locally advanced CRC samples. By constructing a protein–protein interaction network and co-expressing networks, we identified 10 key mRNAs, 4 key lncRNAs, and 7 key snoRNAs. Bioinformatics analysis showed therapy-related mRNAs were associated with nucleosome assembly, chromatin silencing at recombinant DNA, negative regulation of gene expression, and DNA replication. Therapy-related lncRNAs were associated with cell adhesion, extracellular matrix organization, angiogenesis, and sister chromatid cohesion. In addition, therapy-related snoRNAs were associated with DNA replication, nucleosome assembly, and telomere organization. We thought this study provided useful information for identifying novel biomarkers for CRC.

## Introduction

Colorectal cancer (CRC) is one of the most common types of human cancers (Ma et al., 2014). The morbidity and mortality of CRC have increased rapidly in recent years (Budai et al., 2004). In 2016, a total of 134,490 new cases of CRC and 49,190 deaths caused by CRC were reported worldwide. In the past decades, the diagnostic technologies and therapeutic strategies of CRC have made significant progress (Ress et al., 2015). However, the prognosis of CRC remained poor with 5-year survival rates being only 10–15%, and the recurrent disease rates of CRC remained high. Therefore, there was still an urgent need to understand the mechanisms underlying CRC progression and identify novel potential biomarkers for the prognosis of CRC.

Emerging studies had demonstrated that noncoding RNAs played crucial roles in the progression of CRC (Rezanejad Bardaji et al., 2018), including microRNAs, long noncoding RNAs (lncRNAs), and small nucleolar RNA (snoRNAs). The important roles of microRNAs in CRC had been studied clearly (Zhang et al., 2012a). lncRNAs are a large class of transcripts longer than 200 bases, with no protein-coding potential. Previous studies had showed that lncRNAs were associated with CRC progression and prognosis. For example, overexpression of lncRNA TUSC7 reduces cell migration and invasion in CRC (Xu J. et al., 2017). lncRNA KCNQ1OT1 enhanced the methotrexate resistance of CRC cells by regulating miR-760/PPP1R1B (Sunamura et al., 2011). LINC01354 interacting with hnRNP-D contributes to the proliferation and metastasis in CRC through activating Wnt/β-catenin signalling (Zhang et al., 2016). Recent studies have also indicated that snoRNAs were also associated with the progression of CRC, for example, Yoshida et al. (2017).

In the present study, we re-annotated a Gene Expression Omnibus (GEO) dataset GSE94104 to identify CRC-related mRNAs and lncRNAs. Bioinformatics analysis was also performed to understand the potential roles of these lncRNAs in CRC. This study could provide novel clues to prove that CRC-related lncRNAs could serve as biomarkers for CRC.

## Materials and Methods

### lncRNA Classification Pipeline

We used a pipeline described by Zhang et al. to re-annotate microarray data using the following criteria (Zhang et al., 2012b). Briefly, first, GPL570 platform of Affymetrix Human Genome U133 Plus 2.0 Array (Affymetrix Inc., Santa Clara, California, USA) probe set ID was mapped to the NetAffx Annotation Files (HG-U133 Plus 2.0 Annotations, CSV format, release 31, 08/23/10). The annotations included the probe set ID, gene symbol, and Refseq transcript ID. Second, the probe sets that were assigned with a Refseq transcript ID in the NetAffx annotations were extracted. In this study, we only retained those labeled as “NR_” (NR indicates noncoding RNA in the Refseq database). Finally, 2,448 annotated lncRNA transcripts with corresponding Affymetrix probe IDs were generated.

### Microarray Data and Data Preprocessing

By screening colon cancer-related public datasets in GEO database, we selected GSE94104 dataset for further study, which contained the largest number of therapy-related colon cancer samples. In the present study, we downloaded GSE94104 datasets (Tsukamoto et al., 2011) from GEO database to identify differently expressed mRNAs and lncRNAs. A total of 40 matched formalin-fixed paraffin-embedded pre-therapeutic locally advanced rectal cancer biopsy and post-therapeutic locally advanced rectal cancer biopsy samples were included in this study. All samples were provided by the Northern Ireland Biobank and arrayed using the Illumina HumanHT-12 WG-DASL V4 expression beadchip. The raw data were normalized using robust multi-array average method under R 3.4.2 statistical software with affy package from BioConductor. Normalization was separately performed for LCM dataset and homogenized tissue dataset. The normalized gene expression levels were presented as log2-transformed values by robust multi-array average. lncRNAs with fold changes ≥2 and P values <0.05 were considered as differentially expressed lncRNAs.

### Co-Expression Network Construction and Analysis

In this study, the Pearson correlation coefficient of different expressed gene–lncRNA pairs was calculated according to the expression value of them. The co-expressed differentially expressed gene–lncRNA pairs with the absolute value of Pearson correlation coefficient ≥0.6 were selected, and the co-expression network was established by using cytoscape software.

### Functional Group Analysis

The DAVID system (http://david.ncifcrf.gov/) was used to perform Gene Ontology (GO) and Kyoto Encyclopedia of Genes and Genomes (KEGG) pathway enrichment analyses. GO analyses included biological process, cellular component, and molecular function. GO terms and KEGG pathways with a P value of <0.05 were considered as significantly enriched function annotations.

### Protein–Protein Interaction Network and Module Analysis

STRING online software was used to construct a protein–protein interaction (PPI) network (Liu et al., 2009) (https://string-db.org/cgi/input.pl?sessionId=AUH42ZEZwajP&input_page_show_search=on). PPI with the combined score >0.4 was considered as significant. Cytoscape software was used to visualize the PPI network.

## Results

### Transcriptional Analysis of Therapy-Related Messenger RNAs in Pre-Therapeutic Biopsies and Post-Therapeutic Resections of Locally Advanced Colorectal Cancer

**Figure 1:**
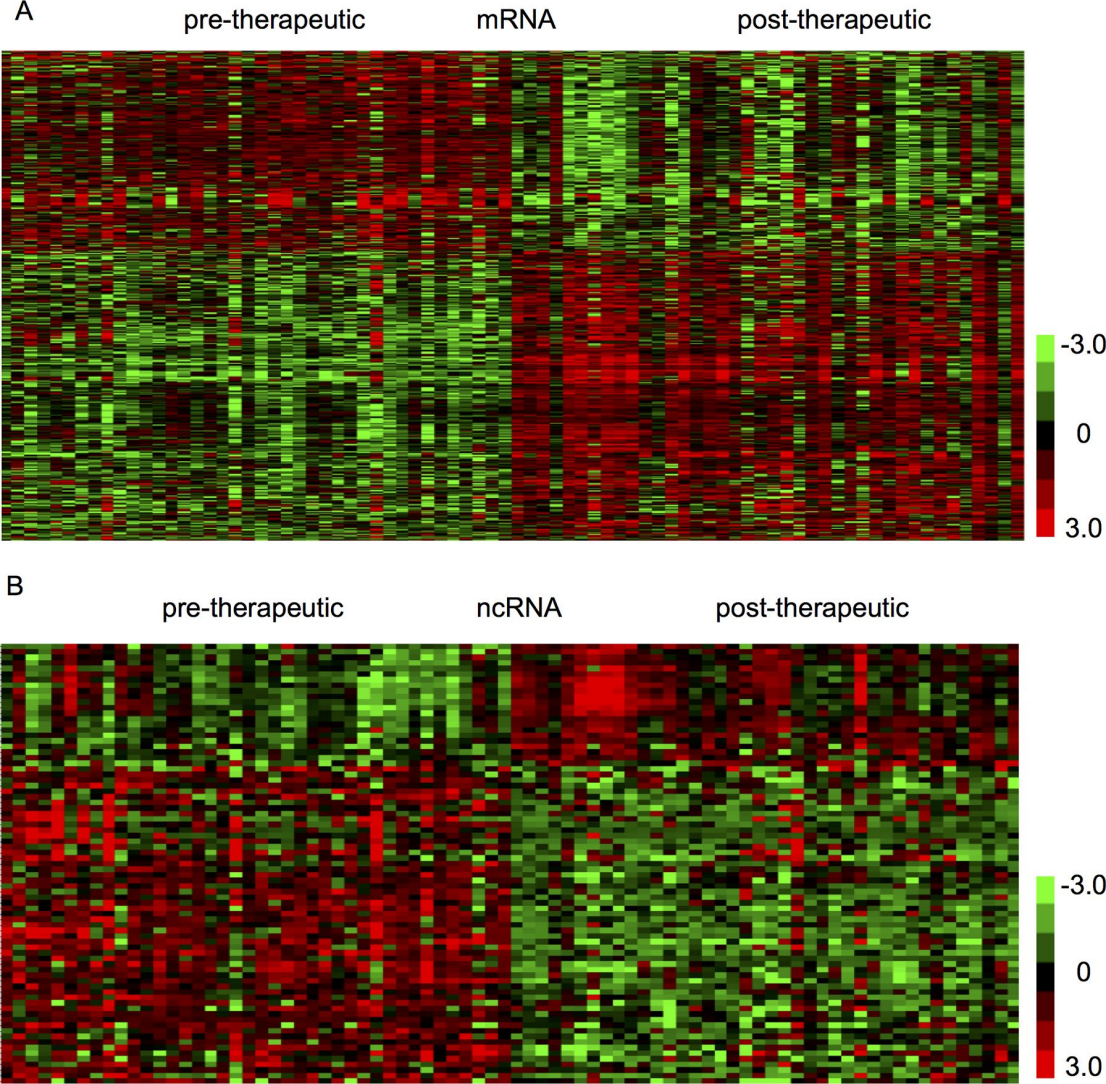
Identification of therapy-related mRNAs and ncRNAs in CRC. **(A)** Hierarchical clustering analysis showed differential mRNAs expression in the CRC by using GSE94104. **(B)** Hierarchical clustering analysis showed differential ncRNAs expression in the CRC by using GSE94104.

The present study aimed to identify therapy-related mRNAs in advanced CRC using a public dataset, GSE94104. A total of 40 pre-therapeutic advanced CRC samples and 40 post-therapeutic advanced CRC samples were included in this dataset. We identified 427 dysregulated mRNAs between pre- and post-therapeutic locally advanced CRC (LACC) samples, including 235 upregulated mRNAs and 192 downregulated mRNAs after therapy in LACC. Hierarchical clustering was used to show differentially expressed mRNAs in post-therapeutic LACC (**Figure 1A**).

### Transcriptional Analysis of Therapy-Related Noncoding RNAs in Pre-Therapeutic Biopsies and Post-Therapeutic Resections of Locally Advanced Colorectal Cancer

Next, we focused on identifying noncoding RNAs between pre- and post-therapeutic LACC samples. A total of 19 snoRNAs and 4 lncRNAs were found to be differently expressed (**Figure 1B**). Among these lncRNAs, we found SERTAD4-AS1 and MIR100HG were upregulated, whereas PCAT18 and KRTAP5-AS1 were downregulated in post-therapeutic LACC samples compared with those in pre-therapeutic LACC samples. Interestingly, we found most of these affected snoRNAs (18/19) were upregulated in post-therapeutic LACC samples compared with those in pre-therapeutic LACC samples, including SNORD116-4, SNORD116-2, SNORD107, SNORD61, SNORD112, SNORD109A, SNORD113-5, SNORD113-8, SNORD113-7, SNORD114-1, SNORD114-11, SNORD113-6, SNORD114-17, SNORD113-9, SNORD113-3, SNORD114-3, SNORD113-2, and SNORD114-13.

### Protein–Protein Interaction Network Analysis of Therapy-Related Messenger RNAs in Locally Advanced Colorectal Cancer

**Figure 2:**
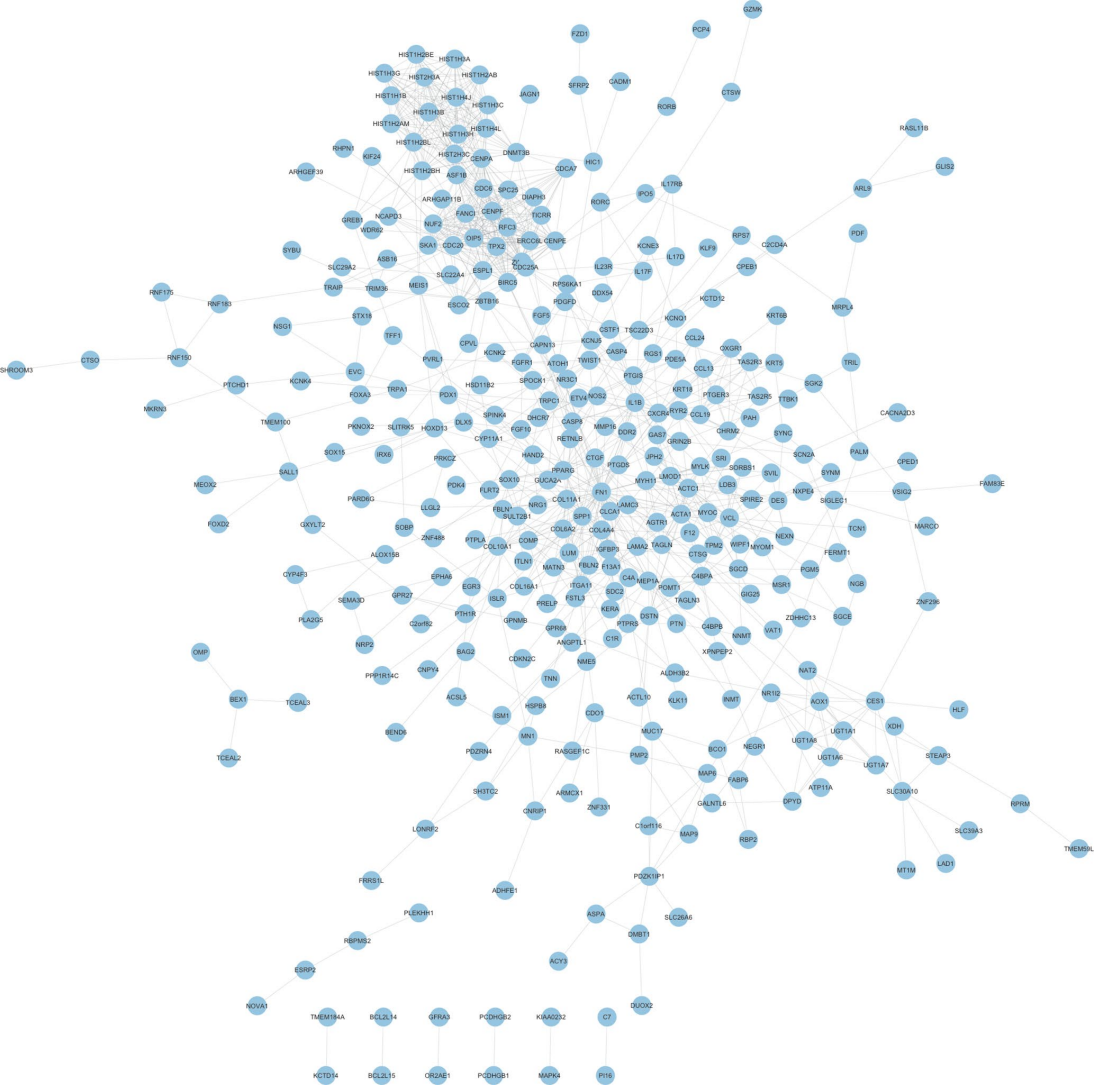
Construction of therapy-related PPI networks in CRC. We constructed therapy-related PPI networks in CRC, including a total of 348 nodes and 1,047 edges.

In order to reveal the relationships among therapy-related mRNAs in LACC, we constructed PPI networks using STRING database. The combined score >0.4 was used as the cutoff criterion. As shown in **Figure 2**, a total of 348 nodes and 1,047 edges were included in this PPI network. The nodes that had higher degrees were identified as hub genes, including FN1, CDC20, SPP1, HIST1H3B, ZWINT, CENPF, HIST1H3C, CXCR4, HIST1H3G, and RFC3.

### Construction of Therapy-Related Long Noncoding RNAs and Small Nucleolar RNAs Regulating Co-Expression Network in Locally Advanced Colorectal Cancer

In order to reveal the potential functions of therapy-related lncRNAs and snoRNAs in LACC, we first performed Pearson correlation calculation between lncRNAs or snoRNAs and mRNAs in LACC. Based on the correlation analysis results, we constructed mRNA–lncRNA/snoRNAs co-expression networks (p-value < 0.05 and absolute value of correlation coefficient >0.7).

**Figure 3:**
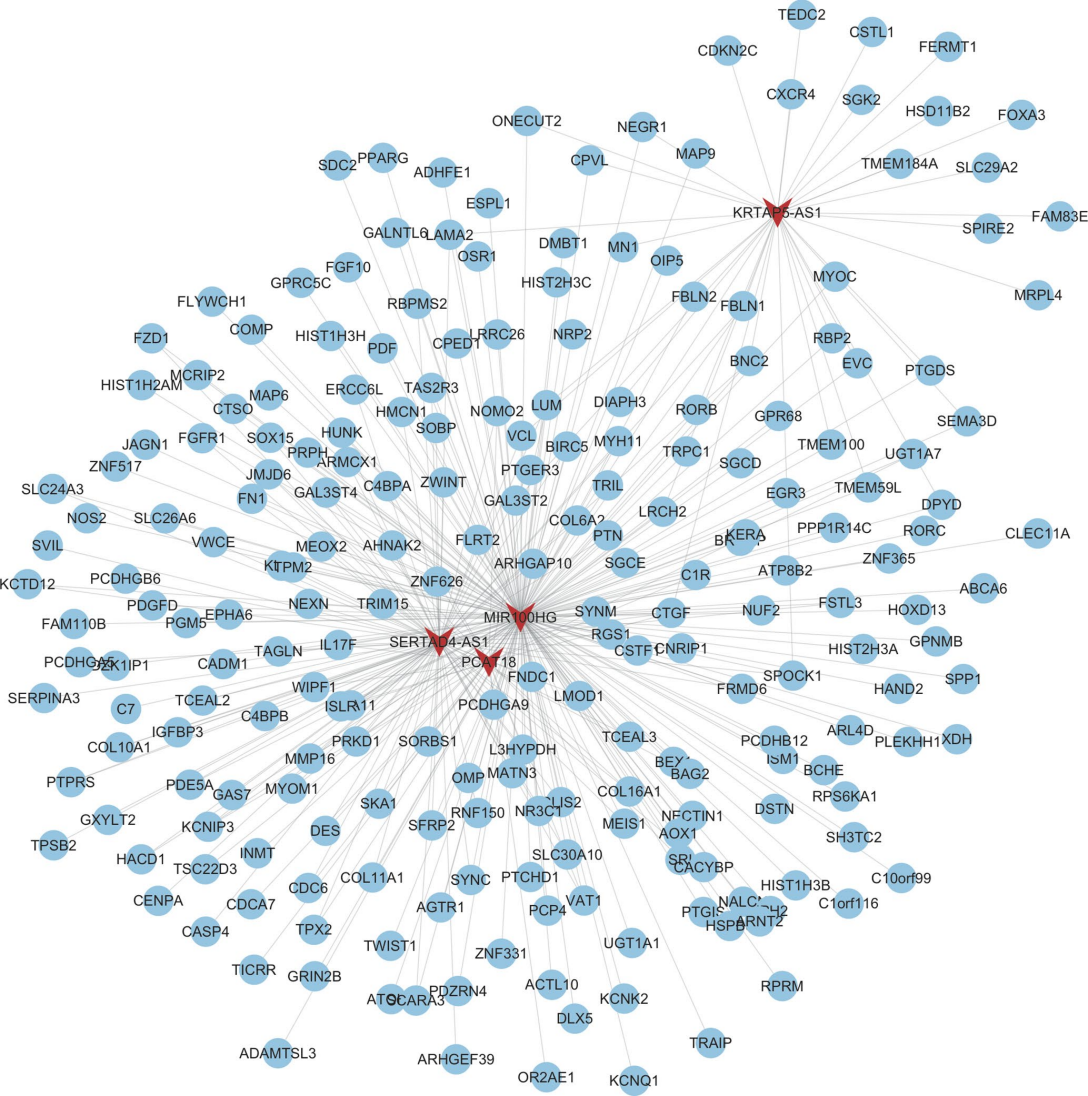
Construction of therapy-related lncRNA regulating co-expression networks in CRC. We constructed therapy-related lncRNA regulating co-expression networks in CRC, including a total of 4 lncRNAs and 226 mRNAs. Red node, lncRNAs; blue nodes, mRNAs.

As shown in **Figure 3**, the mRNAs–lncRNAs co-expression network included 4 lncRNAs (MIR100HG, SERTAD4-AS1, KRTAP5-AS1, and PCAT18) and 226 mRNAs. MIR100HG was the key lncRNA in this network by co-expressing with more than 200 mRNAs, including FZD1, FGFR1, FN1, and KLF9. The top 10 most co-expressing genes of SERTAD4-AS1 included COL16A1, ISLR, ZNF626, COL6A2, SOX15, FRMD6, PCDHGA9, CDC6, TPM2, and C1R. The top 10 most co-expressing genes of KRTAP5-AS1 included DPYD, GAL3ST2, CSTL1, HSD11B2, LRCH2, DIAPH3, FERMT1, MRPL4, NEGR1, and LAMA2. The top 10 most co-expressing genes of PCAT18 included ZNF626, OR2AE1, WIPF1, CTGF, IL17F, L3HYPDH, COL16A1, KCNIP3, PCDHGA9, and COL6A2.

**Figure 4:**
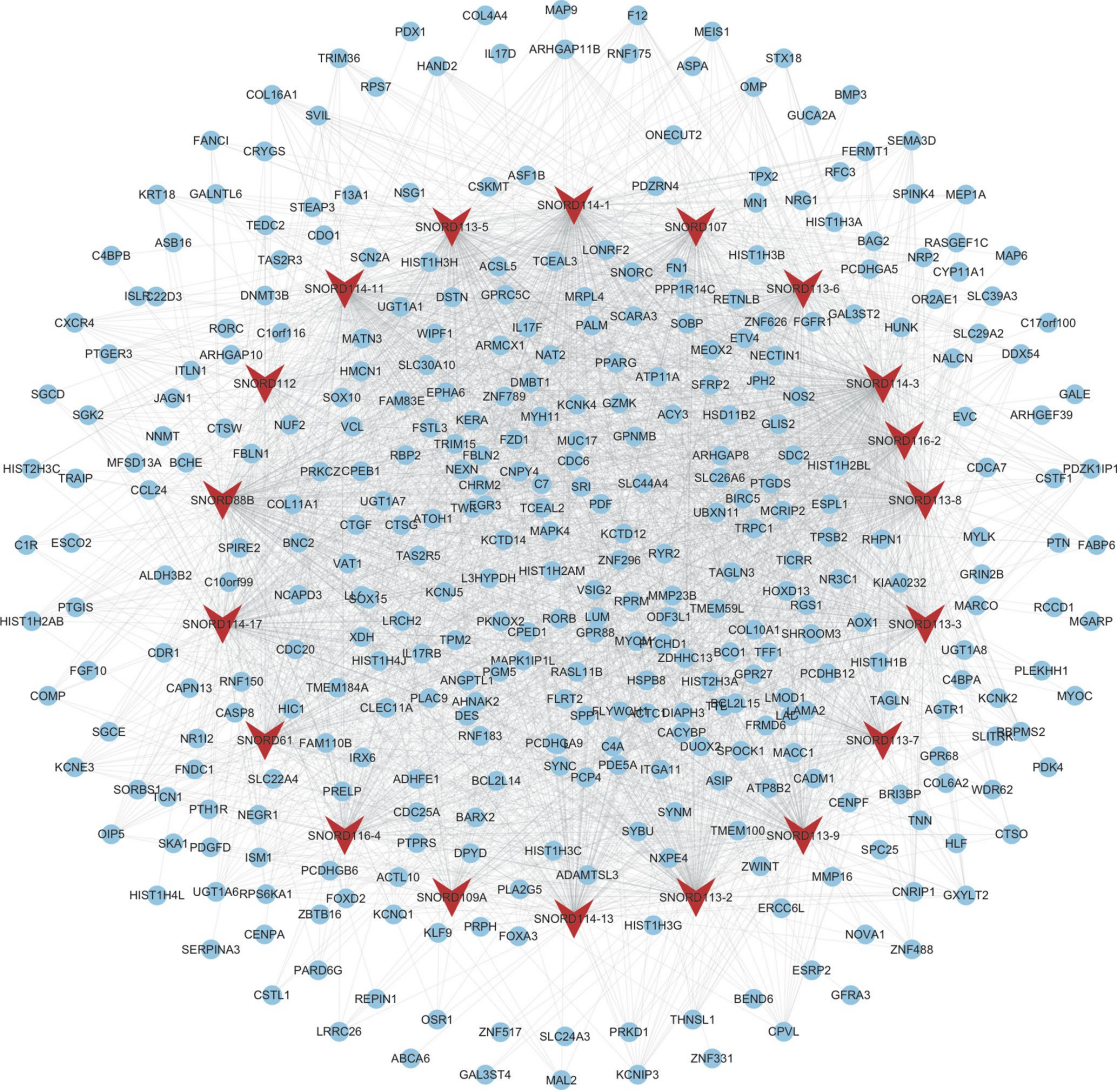
Construction of therapy-related snoRNAs regulating co-expression networks in CRC. We constructed therapy-related snoRNAs regulating co-expression networks in CRC, including a total of 19 snoRNAs and 360 mRNAs. Red node, snoRNAs; blue nodes, mRNAs.

As shown in **Figure 4**, the mRNAs–snoRNAs co-expression network included 19 snoRNAs and 360 mRNAs. Several snoRNAs were identified as key regulators by co-expressing with more than 150 mRNAs, including SNORD114-3, SNORD114-1, SNORD113-5, SNORD88B, SNORD113-8, SNORD114-11, and SNORD113-2.

### Bioinformatics Analysis of Therapy-Related Messenger RNAs in Locally Advanced Colorectal Cancer

**Figure 5:**
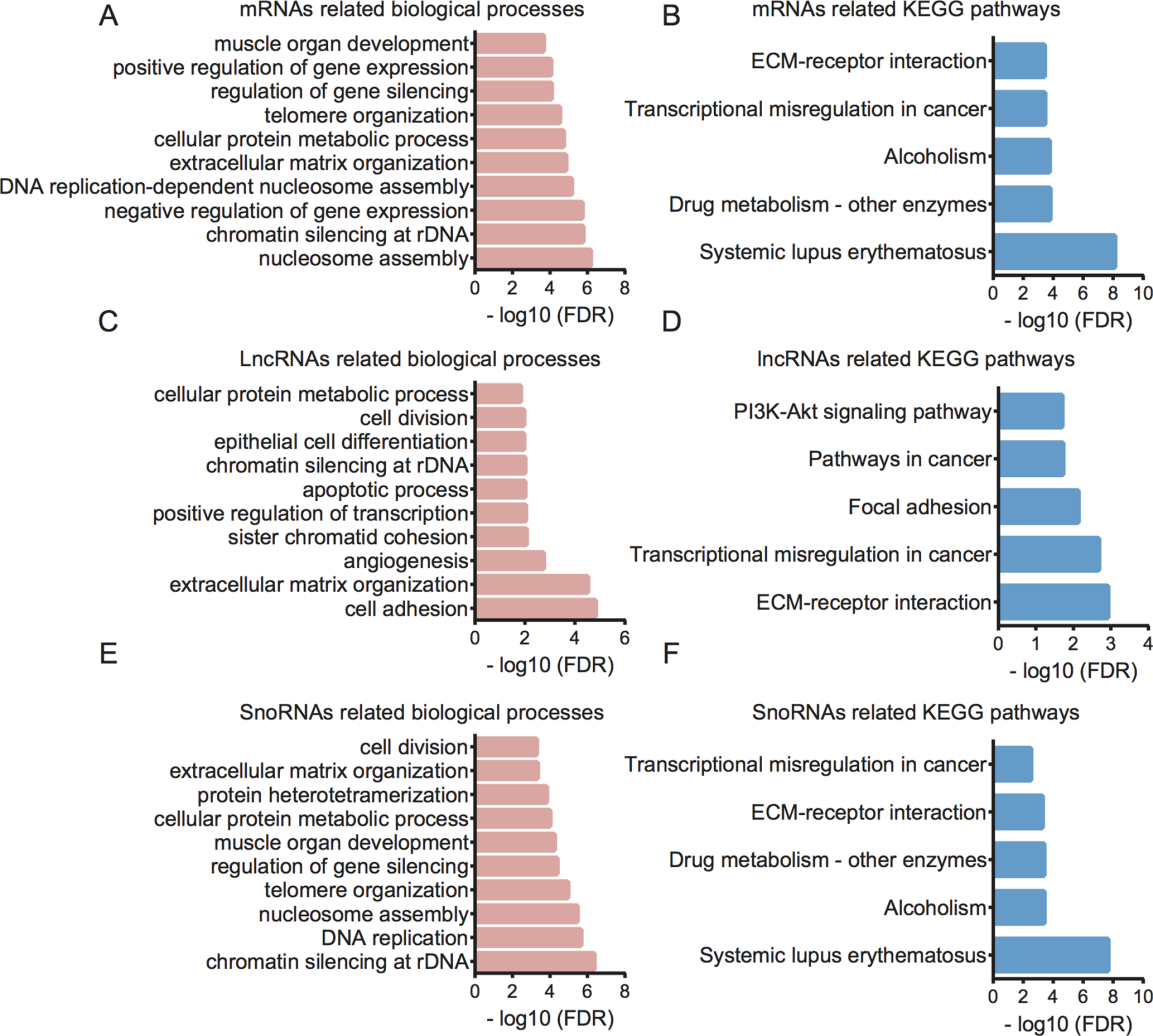
Bioinformatics analysis for therapy-related mRNAs, lncRNAs, and snoRNAs in CRC. **(A)** GO analysis showed therapy-related mRNA-associated biological processes. **(B)** KEGG pathway analysis showed therapy-related mRNA-associated pathways. **(C)** GO analysis showed therapy-related lncRNA-associated biological processes. **(D)** KEGG pathway analysis showed therapy-related lncRNA-associated pathways. **(E)** GO analysis showed therapy-related snoRNA-associated biological processes. **(F)** KEGG pathway analysis showed therapy-related snoRNA-associated pathways.

Furthermore, we performed GO and KEGG analysis for therapy-related mRNAs in LACC (**Figures 5 A, B**). Bioinformatics analysis showed that the therapy-related mRNAs were mainly involved in regulating nucleosome assembly, chromatin silencing at recombinant DNA (rDNA), negative regulation of gene expression, DNA replication-dependent nucleosome assembly, extracellular matrix organization, cellular protein metabolic process, telomere organization, regulation of gene silencing, positive regulation of gene expression, and muscle organ development. KEGG pathway analysis revealed that therapy-related mRNAs were mainly involved in regulating systemic lupus erythematosus, drug metabolism—other enzymes, alcoholism, transcriptional misregulation in cancer, and extracellular matrix (ECM)–receptor interaction.

### Bioinformatics Analysis for Related Long Noncoding RNAs and Small Nucleolar RNAs in Locally Advanced Colorectal Cancer

Then, bioinformatics analysis for related lncRNAs and snoRNAs in LACC was performed using their regulating targets in LACC (**Figures 5 C–F**). GO analysis showed that differentially expressed lncRNAs were associated with cell adhesion, extracellular matrix organization, angiogenesis, sister chromatid cohesion, positive regulation of transcription, apoptotic process, chromatin silencing at rDNA, epithelial cell differentiation, cell division, and cellular protein metabolic process. KEGG pathway analysis indicated therapy-related lncRNAs were associated with ECM–receptor interaction, transcriptional misregulation in cancer, focal adhesion, pathways in cancer, and PI3K-Akt signaling pathway.

GO analysis showed that differentially expressed snoRNAs were associated with chromatin silencing at rDNA, DNA replication, nucleosome assembly, telomere organization, regulation of gene silencing, muscle organ development, cellular protein metabolic process, protein heterotetramerization, extracellular matrix organization, and cell division. KEGG pathway analysis indicated that therapy-related snoRNAs were associated with systemic lupus erythematosus, alcoholism, drug metabolism, ECM–receptor interaction, and transcriptional misregulation in cancer.

### Expression of Key lncRNAs Were Dysregulated in Colorectal Cancer Samples

**Figure 6:**
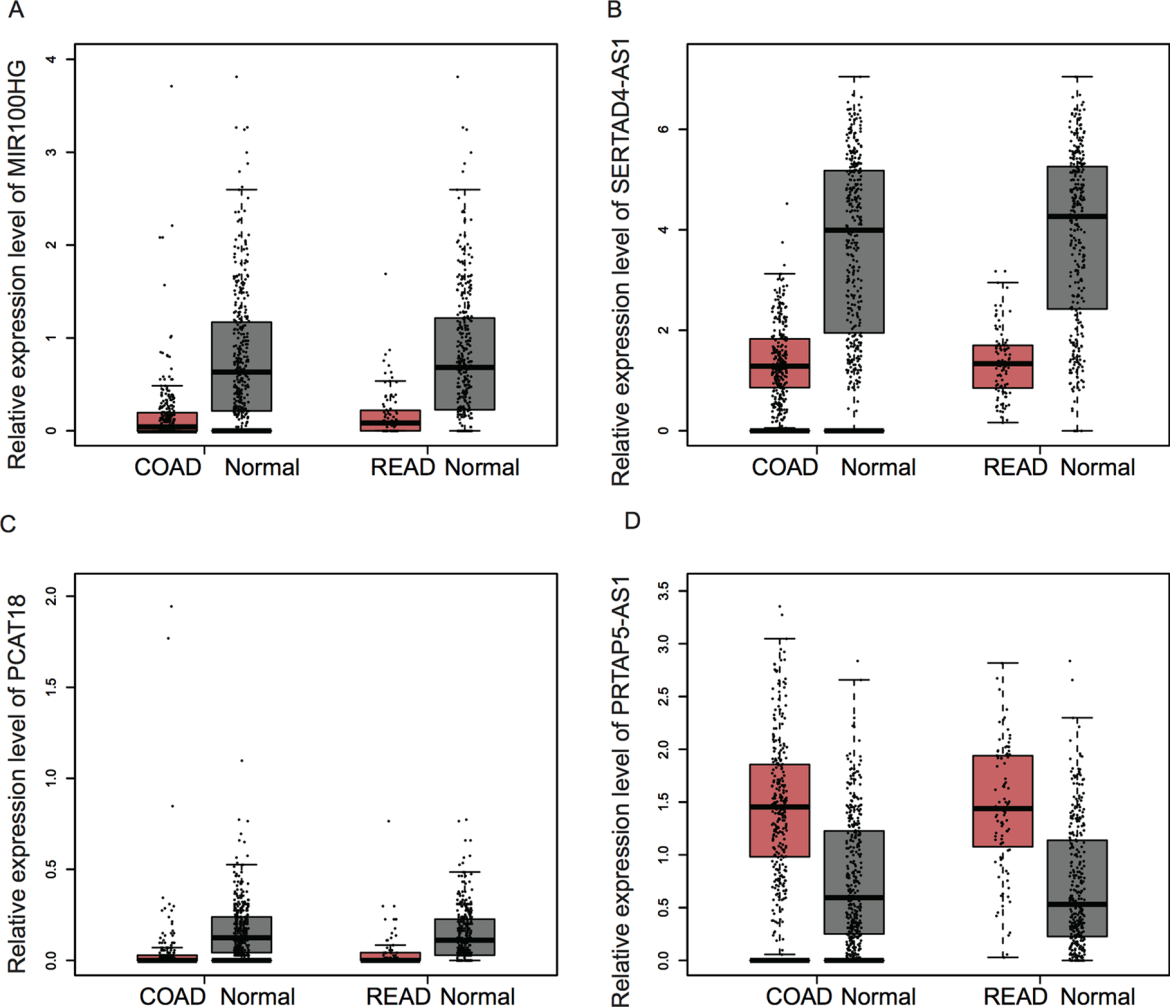
The expression of key lncRNAs were dysregulated in CRC samples. **(A–D)** The expression levels of MIR100HG **(A)**, SERTAD4-AS1 **(B)**, and PCAT18 **(C)** were significantly downregulated; however, KRTAP5-AS1 **(D)** was upregulated in both COAD and READ samples compared with that in normal tissues.

In order to investigate the prognostic value of key lncRNAs in CRC, we analyzed an independent public dataset, the Gene Expression Profiling Interactive Analysis (GEPIA) database. By analyzing the GEPIA database, we found that the expression levels of MIR100HG, SERTAD4-AS1, and PCAT18 were significantly downregulated; however, KRTAP5-AS1 was upregulated in both colon adenocarcinoma (COAD) and rectum adenocarcinoma (READ) samples compared with that in normal tissues (**Figure 6**).

## Discussion

CRC is one of the most common types of human cancer, which is caused by multiple genetic and epigenetic aberrations. However, the mechanisms underlying CRC remained largely unclear. This study identified differently expressed mRNAs, lncRNAs, and snoRNAs between pre-therapeutic biopsies and post-therapeutic resections of locally advanced CRC by analyzing a public dataset, GSE94104. Then, we constructed a PPI network to identify key therapy-related proteins in LACC. Next, we constructed snoRNAs and lncRNAs regulating co-expression networks to identify key therapy-related snoRNAs and lncRNAs in LACC. Finally, GO and KEGG pathway analyses were conducted to predict their potential functions in LACC.

The present study identified a total of 235 upregulated mRNAs and 192 downregulated mRNAs after therapy in LACC. Bioinformatics analysis showed that these mRNAs were associated with nucleosome assembly, chromatin silencing at rDNA, negative regulation of gene expression, and DNA replication. Furthermore, a PPI network including 348 proteins and 1,037 edges were constructed to reveal the relationship among therapy-related proteins. Ten proteins were identified as key regulators in this network, including FN1, CDC20, SPP1, HIST1H3B, ZWINT, CENPF, HIST1H3C, CXCR4, HIST1H3G, and RFC3. FN1 is a novel protein involved in regulating cancer progression (Ifon et al., 2005). FN1 was found to be dysregulated in multiple human cancers, including colon cancer (Cai et al., 2018). In CRC, a single nucleotide polymorphism in FN1 was found to be associated with tumor shape. FN1 was transcriptionally activated by HMGA2, and the suppression of FN1 inhibited CRC growth and metastasis. CDC20 is a key E3 ligase that binds to APC and recognizes D-box or KEN box substrates to promote proteasomal degradation (Paul et al., 2017). CDC20 was frequently overexpressed in malignant tumors, such as prostate cancer, hepatocellular carcinoma, and ovarian cancer. SPP1 was reported to be overexpressed in numerous tumors, such as lung cancer, colon cancer, breast cancer, and prostate cancer (Xu C. et al., 2017). SPP1 was associated with tumor metastasis in gastric cancer and esophageal adenocarcinoma. Zwint is an important regulatory protein for chromosome movement and mitotic checkpoints (Kasuboski et al., 2011). Previous studies have identified Zwint overexpression in breast and ovarian cancers. CENPF is a part of the centromere–kinetochore complex and is a component of the nuclear matrix during G2 of interphase (Sugimoto et al., 1999). Recent studies showed that CENPF played crucial roles in the progression of human cancers. For example, the altered phosphorylation of CENPF affected glutamine uptake in colon cancers (Michalak et al., 2019). CXCR4 is a transmembrane G-protein-couple receptor and played a central role in the neurotropism of cells (Xu et al., 2015). RFC3 was a member of RFC family, which played a key role in DNA replication, DNA damage repair, and checkpoint control. Multiple studies indicated RFC3 was overexpressed and correlated to the progression of human cancers (Shen et al., 2014). These reports together with our findings suggested that these key regulators may play key roles in regulating the therapy-related biological processes in LACC.

Recent studies showed ncRNAs were involved in regulating multiple cancer-related biological processes, such as cell proliferation, apoptosis, and invasion. For example, HAND2-AS1 was observed to suppress CRC proliferation though sponging miR-1275 (Zhou et al., 2018). SNORA21 played as an oncogenic snoRNA in CRC with a prognostic biomarker potential. However, the ncRNAs involved in CRC therapy remained largely unclear. The present study identified 4 lncRNAs and 19 snoRNAs as therapy-related ncRNAs in LACC. Next, lncRNA–mRNA and snoRNAs–mRNA co-expression networks were constructed. Four lncRNAs, including MIR100HG, SERTAD4-AS1, KRTAP5-AS1, and PCAT18, were found to play crucial roles in this progression. KRTAP5-AS1 was reported as a potential biomarker for papillary thyroid carcinoma. *PCAT18* was found to be associated with the progression of gastric cancer (Foroughi et al., 2018) and prostate cancer. For example, PCAT18 silencing inhibited prostate cancer proliferation, migration, and invasion (Zhan et al., 2018). MIR100HG was identified as a key regulator in LACC (Li et al., 2019). A recent study showed that MIR100HG regulates cell cycle by modulating the interaction between HuR and its target mRNAs (Sun et al., 2018). The present study showed that MIR100HG regulated more than 200 mRNAs, including FZD1, FGFR1, FN1, and KLF9. These genes had been demonstrated to be related to CRC progression. For example, KLF9 prevents CRC through inhibition of interferon-related signaling. Downregulation of FN1 suppressed CRC proliferation, migration, and invasion. FZD1 was a key regulator of wnt signaling and involved in regulating CRC metastasis. SERTAD4-AS1 was involved in regulating COL16A1, ISLR, ZNF626, COL6A2, SOX15, FRMD6, PCDHGA9, CDC6, TPM2, and C1R. Among these genes, TPM2 knockdown had been reported to promote CRC progression upon RhoA activation. We also found KRTAP5-AS1 might regulate DPYD, GAL3ST2, CSTL1, HSD11B2, LRCH2, DIAPH3, FERMT1, MRPL4, NEGR1, and LAMA2 in CRC. Among these mRNAs, DPYD variants was reported to be a predictor of 5-fluorouracil toxicity in adjuvant colon cancer treatment. FERMT1 promoted colon cancer metastasis and epithelial–mesenchymal transition progression *via* modulation of β-catenin transcriptional activity. By analyzing the GEPIA database, we found that the expression levels of MIR100HG, SERTAD4-AS1, and PCAT18 were significantly downregulated; however, KRTAP5-AS1 was upregulated in both COAD and READ samples compared with that in normal tissues. Furthermore, we conducted bioinformatics analysis for these therapy-related lncRNAs and snoRNAs. Our results showed therapy-related lncRNAs were associated with cell adhesion, extracellular matrix organization, angiogenesis, and sister chromatid cohesion. In addition, therapy-related snoRNAs were associated with DNA replication, nucleosome assembly, and telomere organization.

Of note, several limitations should be noted in this study. First, the number of samples used in present study were limited. In the further study, more samples should be included to identify therapy-related lncRNAs, snoRNAs, and mRNAs. Second, the detail molecular functions and mechanisms of these key lncRNAs and snoRNAs were unclear. The further validation of these genes should be further investigated. Finally, with the development of next-generation sequence methods, RNA-seq would be a more powerful method to identify novel therapy-related lncRNAs, snoRNAs, and mRNAs in LACC.

In conclusion, we identified 427 dysregulated mRNAs, 4 dysregulated lncRNAs, and 19 dysregulated snoRNAs between pre- and post-therapeutic LACC samples. By constructing a PPI network and co-expressing networks, we identified 10 key mRNAs, 4 key lncRNAs, and 7 key snoRNAs. Bioinformatics analysis showed that therapy-related mRNAs were associated with nucleosome assembly, chromatin silencing at rDNA, negative regulation of gene expression, and DNA replication. Therapy-related lncRNAs were associated with cell adhesion, extracellular matrix organization, angiogenesis, and sister chromatid cohesion. Furthermore, therapy-related snoRNAs were associated with DNA replication, nucleosome assembly, and telomere organization. We think this study provided useful information for identifying novel biomarkers for CRC.

## Data Availability

The datasets generated for this study can be found in the GSE94104.

## Author Contributions

(I) Conception and design: YW, RZ; (II) Administrative support: YW, RZ; (III) Provision of study materials or patients: JL, SM, TL; (IV) Collection and assembly of data: JL, YL, SY; (V) Data analysis and interpretation: JL, WZ; (VI) Manuscript writing: All authors: (VII) Final approval of manuscript: All authors.

## Conflict of Interest Statement

The authors declare that the research was conducted in the absence of any commercial or financial relationships that could be construed as a potential conflict of interest.

## References

[B1] Budai B. Hitre E. Adleff V. Czegledi F. Gyergyay F. Lang I., (2004). The clinical importance of methylenetetrahydrofolate reductase (MTHFR) C677T polymorphism in the 5-fluoropyrimidine-based therapy of metastatic colorectal tumours. Magy Onkola 48, 253–257. HUON.2004.48.3.0253 15520876

[B2] Cai X. Liu C. Zhang T. N. Zhu Y. W. Dong X. Xue P. (2018). Down-regulation of FN1 inhibits colorectal carcinogenesis by suppressing proliferation, migration, and invasion. J. Cell. Biochem. 119 (6), 4717–4728. 10.1002/jcb.26651 29274284

[B3] Foroughi K. Amini M. Atashi A. Mahmoodzadeh H. Hamann U. Manoochehri M. (2018). Tissue-specific down-regulation of the long non-coding RNAs PCAT18 and LINC01133 in gastric cancer development. Int. J. Mol. Sci. 19 (12), 3881. 10.3390/ijms19123881 PMC6321575 30518158

[B4] Ifon E. T. Pang A. L. Johnson W. Cashman K. Zimmerman S. Muralidhar S.(2005). U94 alters FN1 and ANGPTL4 gene expression and inhibits tumorigenesis of prostate cancer cell line PC3. Cancer Cell Int. 5 (1), 19. 10.1186/1475-2867-5-19 15972109 PMC1200560

[B5] Kasuboski J. M. Bader J. R. Vaughan P. S. Tauhata S. B. Winding M. Morrissey M. A. (2011). Zwint-1 is a novel Aurora B substrate required for the assembly of a dynein-binding platform on kinetochores. Mol. Biol. Cell 22 (18), 3318–3330. 10.1091/mbc.e11-03-0213 21775627 PMC3172258

[B6] Li J. Xu Q. Wang W. Sun S. (2019). MIR100HG: a credible prognostic biomarker and an oncogenic lncRNA in gastric cancer. Biosci. Rep. 39 (4). 10.1042/BSR20190171 PMC6449568 30886062

[B7] Liu G. Wong L. Chua H. N. (2009). Complex discovery from weighted PPI networks. Bioinformatics 25, 1891–1897. 10.1093/bioinformatics/btp311 19435747

[B8] Ma Y. Yan F. Li L. Liu L. Sun. J. (2014). Deletion and down-regulation of SMAD4 gene in colorectal cancers in a Chinese population. Chin. J. Cancer Res. 26, 525–531. 10.3978/j.issn.1000-9604.2014.09.02 25400417 PMC4220255

[B9] Michalak M. Warnken U. Schnölzer M. Gabius H. J. Kopitz J. (2019). Detection of malignancy-associated phosphoproteome changes in human colorectal cancer induced by cell surface binding of growth-inhibitory galectin-4. IUBMB Life 71 (3), 364–375. 10.1002/iub.1987 30550624

[B10] Paul D. Ghorai S. Dinesh U. S. Shetty P. Chattopadhyay S. Santra M. K. (2017). Cdc20 directs proteasome-mediated degradation of the tumor suppressor SMAR1 in higher grades of cancer through the anaphase promoting complex. Cell Death Dis. 8 (6), e2882. 10.1038/cddis.2017.270 28617439 PMC5520925

[B11] Ress A. L. Perakis S. Pichler M., microRNAs and Colorectal Cancer, Oxygen Transport to Tissue XXXIII, 889 (2015), 89-103. 10.1007/978-3-319-23730-5_6 26658998

[B12] Rezanejad Bardaji H. Asadi M. H. Yaghoobi M. M. (2018). Long noncoding RNA VIM-AS1 promotes colorectal cancer progression and metastasis by inducing EMT. Eur. J. Cell Biol. 97, 279–288. 10.1016/j.ejcb.2018.04.004 29656793

[B13] Shen H. Cai M. Zhao S. Wang H. Li M. Yao S. (2014). Overexpression of RFC3 is correlated with ovarian tumor development and poor prognosis. Tumour Biol. 35 (10), 10259–10266. 10.1007/s13277-014-2216-2 25030735

[B14] Sugimoto K. Tsutsui M. AuCoin D. Vig B. K. (1999). Visualization of prekinetochore locus on the centromeric region of highly extended chromatin fibers: does kinetochore autoantigen CENP-C constitute a kinetochore organizing center? Chromosome Res. 7 (1), 9–19. 10.1023/A:1009267010071 10219728

[B15] Sun Q. Tripathi V. Yoon J. H. Singh D. K. Hao Q. Min K. W. (2018). MIR100 host gene-encoded lncRNAs regulate cell cycle by modulating the interaction between HuR and its target mRNAs. Nucleic Acids Res. 46 (19), 10405–10416. 10.1093/nar/gky696 30102375 PMC6212728

[B16] Sunamura N. Ohira T. Kataoka M. Inaoka D. Tanabe H. Nakayama Y. (2011). Regulation of functional KCNQ1OT1 lncRNA by beta-catenin. Sci. Rep. 6, 20690. 10.1038/srep20690 PMC4751614 26868975

[B17] Tsukamoto S. Ishikawa T. Iida S. Ishiguro M. Mogushi K. Mizushima H. (2011). Clinical significance of osteoprotegerin expression in human colorectal cancer. Clin. Cancer Res. 17 (8), 2444–2450. 10.1158/1078-0432.CCR-10-2884 21270110

[B18] Xu Q. Wang Z. Chen X. (2015). Stromal-derived factor-1alpha/CXCL12-CXCR4 chemotactic pathway promotes perineural invasion in pancreatic cancer. Oncotarget 6, 4717–4732. 10.18632/oncotarget.3069 25605248 PMC4467110

[B19] Xu C. Sun L. Jiang C. Zhou H. Gu L. Liu Y. (2017a). SPP1, analyzed by bioinformatics methods, promotes the metastasis in colorectal cancer by activating EMT pathway. Biomed. Pharmacother. 91, 1167–1177. 10.1016/j.biopha.2017.05.056 28531945

[B20] Xu J. Zhang R. Zhao J. (2017b). The novel long noncoding RNA TUSC7 inhibits proliferation by sponging MiR-211 in colorectal cancer. Cell. Physiol. Biochem. 41 (2), 635–644. 10.1159/000457938 28214867

[B21] Yoshida K. Toden S. Weng W. Shigeyasu K. Miyoshi J. Turner J. (2017). SNORA21 - An oncogenic small nucleolar RNA, with a prognostic biomarker potential in human colorectal cancer. EBioMedicine 22, 68–77. 10.1016/j.ebiom.2017.07.009 28734806 PMC5552212

[B22] Zhan F. Shen J. Wang R. Wang L. Dai Y. Zhang Y. (2018). Role of exosomal small RNA in prostate cancer metastasis. Cancer Manag. Res. 10, 4029. 10.2147/CMAR.S170610 30319287 PMC6167994

[B23] Zhang P. Ma Y. Wang F. Yang J. Liu Z. Peng J. (2012a). Comprehensive gene and microRNA expression profiling reveals the crucial role of hsa-let-7i and its target genes in colorectal cancer metastasis. Mol. Biol. Rep. 39 (2), 1471–1478. 10.1007/s11033-011-0884-1 21625861

[B24] Zhang X. Sun S. Pu J. K. Tsang A. C. Lee D. Man V. O. (2012b). Long non-coding RNA expression profiles predict clinical phenotypes in glioma. Neurobiol. Dis. 48 (1), 1–8. 10.1016/j.nbd.2012.06.004 22709987

[B25] Zhang Z. Zhou C. Chang Y. Zhang Z. Hu Y. Zhang F. (2016). Long non-coding RNA CASC11 interacts with hnRNP-K and activates the WNT/beta-catenin pathway to promote growth and metastasis in colorectal cancer. Cancer Lett. 376 (1), 62–73. 10.1016/j.canlet.2016.03.022 27012187

[B26] Zhou J. Lin J. Zhang H. Zhu F. Xie R. (2018). LncRNA HAND2-AS1 sponging miR-1275 suppresses colorectal cancer progression by upregulating KLF14. Biochem. Biophys. Res. Commun. 503 (3), 1848–1853. 10.1016/j.bbrc.2018.07.125 30078677

